# Polycystic Ovary Syndrome Revisited: Novel Insights and Updates

**DOI:** 10.7150/ijms.119968

**Published:** 2026-01-01

**Authors:** Wenwen Zhao, Jiayi Zhou, Yanhua Song, Miao He, Xudong Zhu, Bianfang Yu, Wenxiao Gao

**Affiliations:** 1 Health Management Center, Affiliated Hospital of Shandong University of Traditional Chinese Medicine, Jinan 250011, China.; 2 Basic Medical College, Liaoning University of Traditional Chinese Medicine, Shenyang 110847, China.; 3 Medical Insurance Office, Cancer Hospital of Dalian University of Technology, Cancer Hospital of China Medical University, Liaoning Cancer Hospital & Institute, Shenyang, Liaoning 110042, China.; 4 Department of General Surgery, Cancer Hospital of Dalian University of Technology, Cancer Hospital of China Medical University, Liaoning Cancer Hospital & Institute, Shenyang, Liaoning 110042, China.; 5 Department of Anorectal, Affiliated Hospital of Shandong University of Traditional Chinese Medicine, Jinan 250011, China.; 6 Department of Obstetrics, Affiliated Hospital of Shandong University of Traditional Chinese Medicine, Jinan 250011, China.

**Keywords:** polycystic ovary syndrome, environmental factors, pathogenesis, neurotransmitters, therapeutic strategies

## Abstract

Polycystic ovary syndrome (PCOS) is a common endocrine disorder affecting approximately 10% of middle-aged women worldwide. The main clinical features of PCOS include hirsutism, anovulation, irregular menstruation, and polycystic ovaries. However, the exact etiology and underlying mechanisms of PCOS remain incompletely understood. Increasing evidence suggests that a variety of factors—including environmental toxins, chronic low-grade inflammation, oxidative stress, and dysregulated insulin secretion—are involved in the onset and progression of PCOS. Additionally, abnormalities in neurotransmitter metabolism play a significant role in the pathophysiology of PCOS, particularly in relation to weight gain, hyperandrogenemia, and mood disorders. Patients with PCOS often exhibit neuroendocrine system dysfunction, characterized by altered levels of neurotransmitters such as serotonin, norepinephrine, and dopamine. These changes are closely associated with clinical symptoms such as anxiety, depression, obesity, and metabolic disturbances. Current treatment strategies for PCOS primarily focus on lifestyle modifications and pharmacological interventions. These include dietary changes, nutritional supplementation, physical activity, and ovulation induction, all aimed at alleviating symptoms and improving quality of life. Notably, interventions targeting neurotransmitter metabolism are emerging as a novel area of research. The use of antidepressants, anxiolytics, and insulin-sensitizing agents has shown potential in alleviating symptoms and regulating neuroendocrine function. Furthermore, PCOS is significantly associated with metabolic dysfunction, particularly in hormone metabolism, lipid metabolism, and glucose metabolism, which exacerbates the risk of obesity, hyperandrogenemia, and type 2 diabetes. Therefore, understanding alterations in neurotransmitter metabolism and developing new therapeutic strategies may offer novel perspectives and intervention approaches for the diagnosis and treatment of PCOS.

## 1. Background

Polycystic ovary syndrome (PCOS) is a multifactorial endocrine disorder influenced by genetic predisposition, hormonal imbalances, and environmental exposures, affecting approximately 10% of women globally. It poses significant challenges to reproductive health, dermatological conditions such as acne, and psychological well-being—often with devastating consequences for women of reproductive age 1, 2. According to estimates released by the World Health Organization (WHO) in 2023, PCOS affects between 6% and 13% of women of childbearing age. Alarmingly, approximately 70% of affected individuals remain undiagnosed, preventing timely intervention and management.

Diagnosis of PCOS requires adherence to the Rotterdam ESHRE/ASRM criteria, which include oligovulation or anovulation, hyperandrogenism, and polycystic ovarian morphology on ultrasound examination (≥20 follicles measuring 2-9 mm per ovary or ovarian volume ≥10 cm³, based on the 2018 international evidence-based guideline) 3, 4. The pathogenesis of PCOS is complex and heterogeneous, involving intrauterine developmental abnormalities, obesity, and metabolic syndrome 4. Furthermore, advanced glycation end products and steroid hormones contribute to insulin resistance and elevated circulating insulin levels, which in turn stimulate excessive androgen production by the ovaries. This hormonal imbalance disrupts hepatic synthesis of sex hormone-binding globulin (SHBG), leading to further exacerbation of the pathophysiological processes associated with PCOS 5.

Despite increasing research attention and financial support in recent years, the precise etiology and underlying mechanisms of PCOS remain unclear and widely debated 6. Studies indicate that obese women are at a higher risk for developing PCOS and face an increased likelihood of experiencing more severe metabolic and reproductive complications. However, it is important to note that a substantial proportion (approximately 40-50%) of women with PCOS are lean or non-obese, and these individuals may present with distinct clinical and metabolic profiles. Concurrently, altered expression of various genes highlights the significant roles of genetic factors in the development of PCOS. Several studies have demonstrated that obese women are at increased risk of developing PCOS and tend to experience more severe complications 7. Simultaneously, alterations in gene expression further underscore the importance of hereditary factors in PCOS pathophysiology 8. This is especially evident among first-degree relatives of PCOS patients, who are more likely to develop hyperandrogenism and type 2 diabetes. Familial clustering of abnormal gene expression has also been shown to correlate with elevated PCOS risk, reinforcing the significance of genetic influence 9.

The adverse consequences of PCOS are largely attributable to dysregulated hormone metabolism. Insulin resistance, in particular, impairs glucose utilization in peripheral tissues, leading to obesity and exacerbation of PCOS-related symptoms 10. In addition, PCOS is often associated with disordered eating behaviors, such as binge eating, which further promote fat accumulation and contribute to the onset of psychological conditions, including anxiety. These mental health issues may ultimately progress to depression and cognitive decline, complicating the clinical picture 11.

Current clinical strategies for PCOS management primarily emphasize weight control and mental health support, aiming to relieve symptoms and minimize the risk of psychological comorbidities 12. In recent years, there has been growing interest in complementary therapies, including vitamin and mineral supplementation, acupuncture, and yoga, which are increasingly adopted by patients to manage symptoms and improve overall health. These approaches are favored for their relatively low risk of side effects and high safety profiles 13.

Beyond the traditional understanding of PCOS, recent investigations have begun to explore the potential roles of neurotransmitter dysregulation in its pathogenesis. This includes the involvement of gonadotropin-releasing hormone (GnRH), acetylcholine, and dopamine. Emerging evidence suggests that overactivation of GnRH, reduced acetylcholine responsiveness, and dopaminergic imbalances may contribute not only to the physiological manifestations of PCOS but also to its psychological complications. This evolving perspective provides a broader framework for understanding PCOS and opens new avenues for targeted therapeutic interventions. In particular, both pharmacological and non-pharmacological treatments aimed at modulating neurotransmitter activity—such as antidepressants, anxiolytics, acupuncture, and yoga—have shown promise in alleviating symptoms and enhancing fertility outcomes.

## 2. Pathogenic mechanisms of PCOS

Numerous clinical and basic studies have identified hyperandrogenism, insulin resistance, and obesity as the primary contributors to PCOS (Details are summarized and presented in **Table 1**). However, the precise underlying mechanisms remain unclear 14.

Clinical studies in humans have demonstrated several key pathogenic factors. Obesity is a hallmark feature of PCOS, with excessive fat accumulation often cited as a key factor in its development. In obese PCOS patients, the progressive accumulation of adipose tissue, particularly visceral fat, elevates blood fatty acid levels, which in turn promotes hyperglycemia. This leads to hyperinsulinemia, stimulates the luteinizing hormone (LH) receptor, and subsequently increases the luteinizing hormone/follicle stimulating hormone ratio. This cascade of events disrupts follicular development and ovulation, resulting in the formation of polycystic ovaries 15.

Insulin resistance has been established as an independent factor in the onset of PCOS in clinical populations. Insulin regulates the production of various hormones, notably androgens, and influences the development of ovarian cystic structures and the activation of P450c17 enzymes. As a key modulator in steroid hormone synthesis, P450c17 enzymes are integral to human steroidogenesis. Their steroid hydroxylation and carbon-carbon bond cleavage activities facilitate the biosynthesis of glucocorticoids and androgens. This interplay contributes to elevated androgen levels and their accumulation in the body. Additionally, insulin reduces the concentration of sex hormone-binding globulin, leading to increased ovarian testosterone levels. Insulin also promotes lipogenesis while inhibiting lipolysis, thereby contributing to adipose tissue accumulation, which can further promote the development of PCOS 16.

Insights from animal models have provided additional mechanistic understanding of PCOS pathogenesis. Studies in experimental models suggest that leptin may play a role in the pathogenesis of PCOS. As a peptide hormone, leptin primarily regulates energy metabolism and inhibits aromatase expression in granulosa cells, thus reducing the conversion of androgens to estrogens, which may contribute to excessive androgen accumulation 17. Furthermore, research in animal models suggests that the onset and progression of PCOS may be associated with aberrant expression of specific proteases during ovulation, particularly a disintegrin and metalloproteinase with thrombospondin motifs 1 (ADAMTS-1), a member of the metalloproteinase family activated by the LH peak. ADAMTS-1 is crucial in the ovulation process, especially prior to ovulation, when its increased expression leads to extracellular matrix degradation and follicular rupture, releasing the oocyte. Inhibition of this enzyme in experimental studies results in ovarian stromal expansion and the formation of numerous large follicles, culminating in polycystic ovaries 18.

These factors significantly influence the onset and progression of PCOS based on both clinical observations and experimental studies, although further research is needed to elucidate the precise pathogenic mechanisms underlying the disorder and to validate findings from animal models in human populations.

## 3. Correlations between the clinical symptoms of PCOS and abnormal neurotransmitter metabolism

PCOS is one of the leading causes of infertility in women of reproductive age. A growing body of evidence suggests that the development of PCOS is closely associated with dysregulation of neurotransmitter metabolism, including gamma-aminobutyric acid (GABA), glutamate, dopamine, and acetylcholine. These neurotransmitters play important roles in the neuroendocrine regulation of PCOS. Imbalances in neurotransmitters often result in significant psychological consequences for patients with PCOS. Moreover, studies have shown that the incidence of depression in women with PCOS is significantly higher than in the general population, and the risks of anxiety disorders and eating disorders are also markedly increased 19. Therefore, the abnormal alterations in neurotransmitter levels in PCOS warrant special attention. Modulating neurotransmitter expression in these patients may hold therapeutic potential for alleviating clinical symptoms.

### 3.1 Gonadotropin-releasing hormone (GnRH) and the neuroendocrine axis in PCOS

In women of reproductive age, PCOS is frequently accompanied by infertility and elevated levels of luteinizing hormone (LH). The core mechanism underlying this dysfunction lies in abnormal pulsatile secretion of hypothalamic gonadotropin-releasing hormone (GnRH). Substantial evidence indicates that hyperactivity of GnRH neurons is a key driver of neuroendocrine disturbances in PCOS. Additionally, compared to reproductive-age women without fertility issues, PCOS patients consistently exhibit significantly higher anti-Müllerian hormone (AMH) levels both in non-pregnant states and throughout pregnancy.

#### 3.1.1 The central role and regulatory mechanisms of GnRH dysregulation

Women of reproductive age with PCOS often experience infertility, accompanied by elevated LH levels. Dysregulated GnRH pulsatility from the hypothalamus drives excessive LH secretion by the anterior pituitary through the hypophyseal portal system. The resultant hyperandrogenism and altered LH:FSH ratio disrupt follicular development, ovulation, and endometrial receptivity, thereby impairing fertilization and embryonic implantation. Animal studies have confirmed that elevated AMH can induce maternal ovulatory dysfunction and polycystic ovary-like abnormalities, and even trigger similar neuroendocrine phenotypes in the offspring during adulthood. Treatment with GnRH antagonists significantly alleviates these symptoms 20..

**Figure 1:**
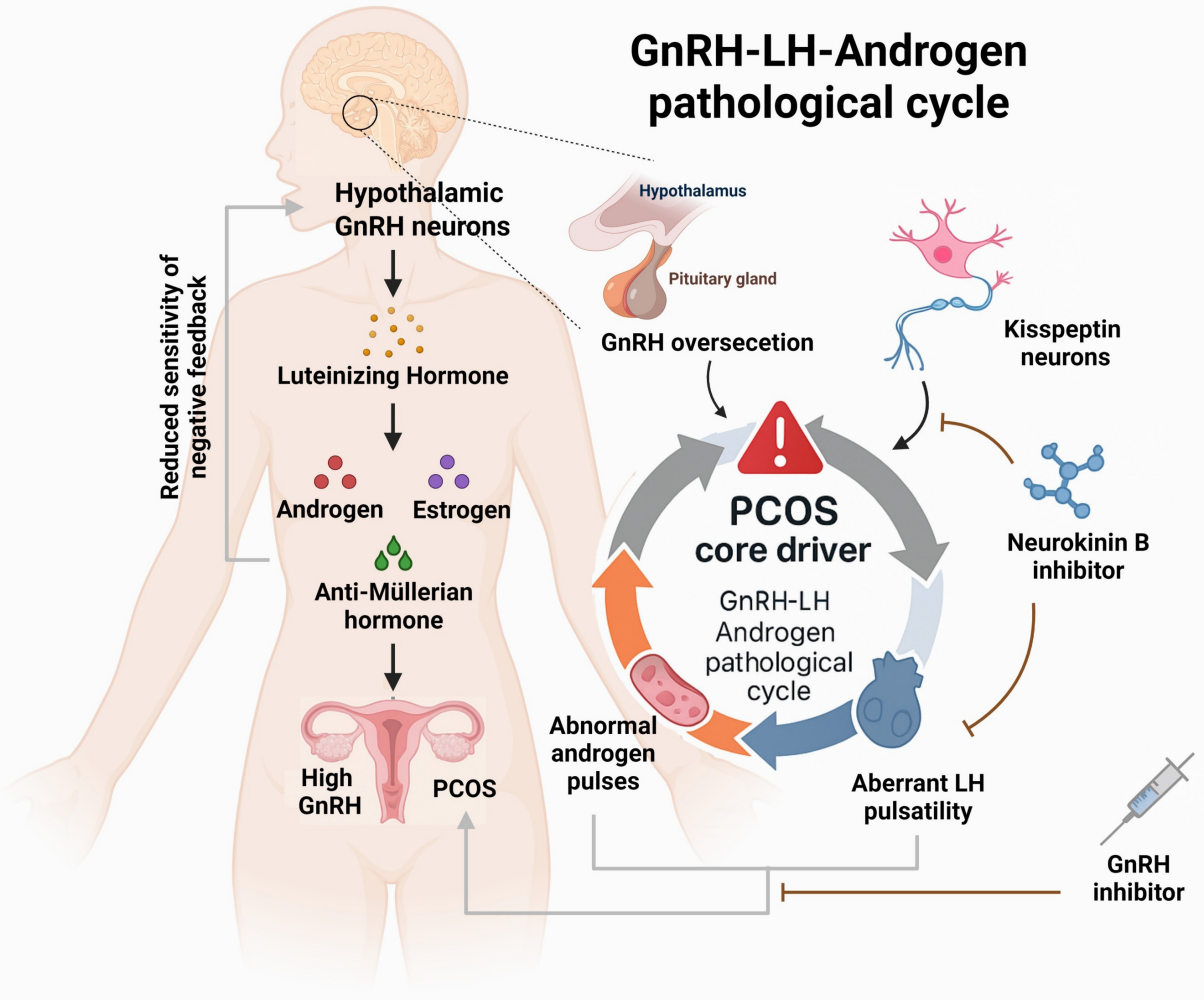
** The relationships between the clinical symptoms of PCOS and the metabolism of gonadotropin-releasing hormone. Left:** Illustrates the pathological cascade of the hypothalamic-pituitary-ovarian axis, with the fundamental etiology being failure of negative feedback due to diminished sensitivity to sex steroid hormones. **Right:** Highlights the therapeutic significance of the kisspeptin-neurokinin B signaling axis within this vicious cycle. Neurokinin B inhibitors effectively suppress pulsatile gonadotropin-releasing hormone release triggered by kisspeptin neurons, thereby reducing luteinizing hormone and androgen levels, consequently alleviating clinical manifestations of PCOS.

Further research has revealed that the elevated GnRH in PCOS is primarily due to reduced sensitivity to negative feedback from sex hormones such as estrogen and progesterone. This leads to increased secretion of LH and androgens, forming a self-reinforcing pathological feedback loop. Disrupting this loop is considered an effective strategy for alleviating the clinical manifestations of PCOS 21. To test this hypothesis, one study employed a chemogenetic approach using clozapine-N-oxide to specifically activate GnRH neurons in mice. The results showed that female mice exhibited neuroendocrine disturbances, abnormal hormone secretion, disruption of estrous cyclicity, and an increased number of ovarian follicles—hallmarks of a PCOS-like phenotype. These findings provide direct support for the hypothesis that hyperactivity of GnRH neurons is a major cause of hormonal imbalance and ovarian dysfunction in PCOS 22. Collectively, these studies demonstrate that dysregulated GnRH expression is closely linked to the clinical features of PCOS and represents a central mechanism in its pathogenesis (**see Figure 1**).

#### 3.1.2 Upstream regulation of GnRH secretion by kisspeptin

Kisspeptin, encoded by the *Kiss1* gene, is a critical upstream regulator of GnRH neurons. By binding to its receptor GPR54, it directly stimulates GnRH secretion and serves as the core initiating signal of the hypothalamic-pituitary-gonadal (HPG) axis. Clinical observations have shown that serum kisspeptin levels are significantly elevated in PCOS patients compared to healthy individuals, suggesting its active involvement in disease progression 23. In fact, kisspeptin neurons are considered to be the "pulse generator" for GnRH secretion 24. Interventional studies targeting this pathway offer new therapeutic prospects. It has been shown that inhibition of neurokinin B (NKB), which is co-expressed in kisspeptin neurons, can attenuate GnRH pulse generation, thereby reducing LH and androgen levels 25. In a clinical trial, PCOS patients who received a neurokinin B receptor antagonist for one month experienced a significant reduction in both LH and androgen concentrations 26. These findings confirm the therapeutic potential of modulating the kisspeptin signaling pathway to correct the excessive GnRH/LH secretion observed in PCOS.

### 3.2 The relationship between PCOS clinical symptoms and acetylcholine metabolism

Multiple studies have identified a higher prevalence of irritable bowel syndrome (IBS) among patients with PCOS 27, although the exact mechanisms underlying abnormal gastrointestinal (GI) motility remain unclear. Wanger et al. established a rat model of PCOS using dihydrotestosterone and euthanized the rats after 17 weeks to evaluate their gastrointestinal response to acetylcholine. The results showed that the frequency and contractility of acetylcholine responses in the gastric fundus and corpus were significantly reduced in PCOS rats. Additionally, colonic smooth muscle contractility and responsiveness to exogenous calcium ions were also diminished. A reduction in the phosphorylation of 20 kDa myosin light chain (MLC20) was also observed in colonic tissue. MLC20 plays a key role in muscle contraction, cell division, and apoptosis by regulating the activity and conformation of myosin 28, 29.

Taken together, these findings suggest that PCOS may impair gastrointestinal contractility and induce gastroparesis by reducing acetylcholine responsiveness 27. One study assessed the therapeutic effect of *Wuxinghuafang*, a traditional Chinese herbal formula, in a PCOS rat model. It was found that lysine, ornithine, and acetylcholine levels were elevated in the hyperinsulinemia group. These results suggest that *Wuxinghuafang* may alleviate PCOS symptoms by modulating inflammation and oxidative stress 30.

**Figure 2:**
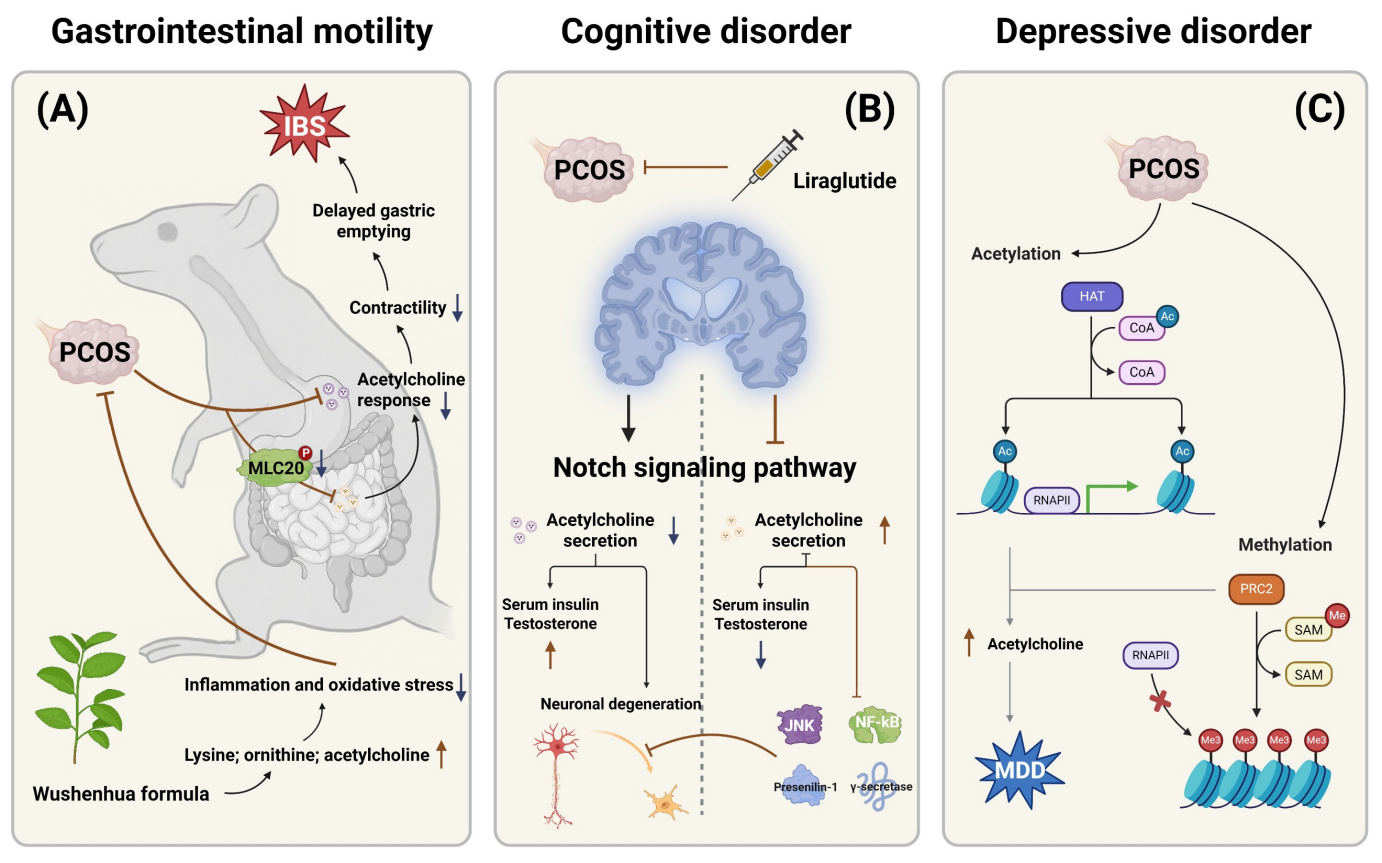
** The relationships between the clinical symptoms of PCOS and acetylcholine metabolism.** This figure systematically summarizes the mechanisms underlying the tripartite pathological manifestations of PCOS involving gastrointestinal, neurological, and psychiatric systems in relation to acetylcholine metabolism, highlighting the pivotal roles of acetylcholine dysregulation in PCOS pathogenesis. (A) Gastrointestinal Motility Disorders and Therapeutic Interventions in PCOS. (B) Notch Signaling-Mediated Cognitive Dysfunction in PCOS. (C) Epigenetically Regulated Depressive-like Behaviors in PCOS.

Endocrine dysregulation in PCOS has also been implicated in cognitive impairment. Recent studies have proposed that the Notch signaling pathway may play a role in both the pathogenesis of PCOS and various neurodegenerative diseases. Liraglutide, a Notch pathway inhibitor, has shown neuroprotective properties and potential in preventing cognitive decline 31. A study exploring the effects of liraglutide on PCOS rats found that the Notch signaling pathway was markedly activated in the control group, accompanied by neuronal degeneration. In contrast, the experimental group showed significant inhibition of the Notch pathway, improvement in memory deficits, and decreased expression of JNK and *Presenilin-1* genes, along with reduced levels of pro-inflammatory transcription factor NF-κB and γ-secretase. Moreover, liraglutide significantly lowered testosterone and insulin levels in PCOS rats 32. The incidence of depression is notably high in PCOS patients, with studies suggesting a potential link to histone deacetylation and DNA methylation, particularly in severe cases of depression 33. Acetylcholine is also implicated in this relationship. John et al. demonstrated that administering letrozole orally for 21 days to wild-type female Wistar rats induced symptoms such as hyperandrogenism, ovarian cyst formation, vesicle degeneration, and depressive behavior. Furthermore, elevated DNA methyltransferase expression was observed in the hippocampus, and biochemical analysis revealed significant increases in malondialdehyde and acetylcholine levels, along with neurodegeneration 33. These observations highlight the connection between depression in PCOS patients and neuroinflammation, as well as the upregulation of acetylcholine. Additionally, acetylcholine appears to have a dual role in PCOS progression, with its exact mechanism requiring further investigation. The relationships between the clinical symptoms of PCOS and acetylcholine metabolism were also summarized in **Figure 2**.

### 3.3 The relationship between PCOS clinical symptoms and dopamine metabolism

**Figure 3:**
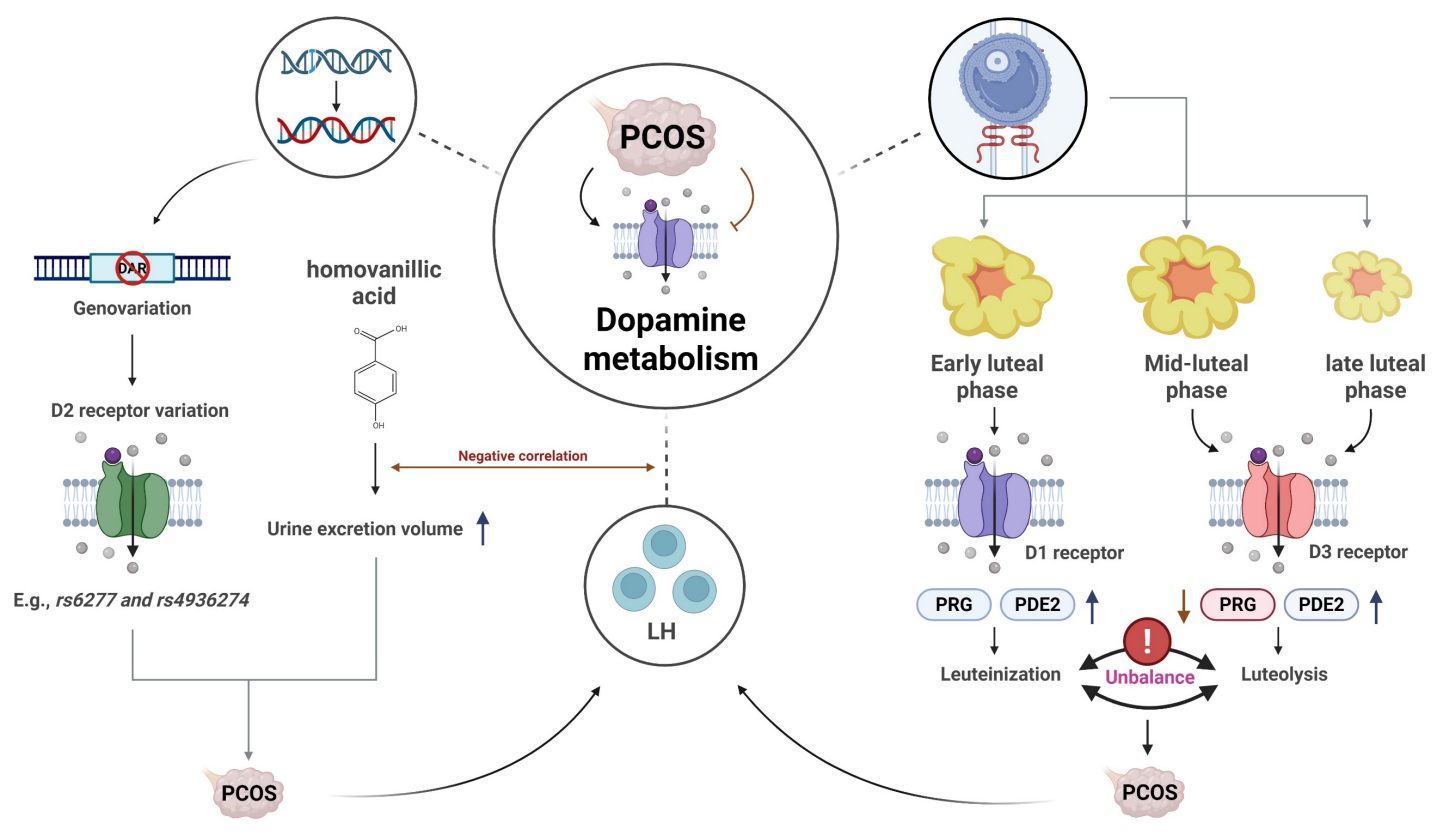
** The association between clinical symptoms of PCOS and dopamine metabolism.** This figure sequentially illustrates the pathological mechanisms linking PCOS to dopamine metabolism, encompassing genetic susceptibility conferred by DRD2 variants, significantly elevated urinary excretion of the dopamine metabolite HVA, and biphasic regulation of luteal function mediated by dopamine receptors.

Dopamine, a neurotransmitter primarily located in the brain, exerts its effects by binding to G-protein-coupled receptors 34. Studies have suggested that dopamine dysregulation may be associated with ovarian dysfunction in PCOS patients. In vivo studies on pseudopregnant rabbits revealed that dopamine and D1 receptor agonists promoted the release of progesterone (PRG) and prostaglandin E2 (PGE2) during the early luteal phase. In contrast, dopamine and D3 receptor agonists were shown to reduce progesterone levels and increase PGE2 release. These findings suggest that dopamine/D1 receptor interactions may promote luteal formation, whereas dopamine/D3 receptor interactions may facilitate luteal regression, providing a novel theoretical framework for understanding luteal dysfunction in PCOS 35. One study compared body mass index (BMI), blood glucose, LH, dopamine, and prolactin levels between PCOS patients and healthy controls. The results showed that BMI was significantly higher in PCOS patients, along with elevated levels of prolactin, dopamine, and LH—all of which were positively correlated with the presence of PCOS 36. These findings indirectly suggest that excessive dopamine expression may play an important role in the progression of PCOS. Another study further emphasized the role of dopamine in the pathogenesis of PCOS. Dopamine receptor D2 (DRD2) is known to inhibit excitatory input to GnRH neurons and suppress prolactin secretion. Therefore, *DRD2* gene polymorphisms may contribute to the maintenance of PCOS progression. Researchers analyzed 22 DRD2 gene variants in Italian families and found that five novel variants (e.g., rs6277 and rs4936274) were significantly associated with increased PCOS risk, although further validation with larger sample sizes is required 8. Additionally, the measurement of homovanillic acid (HVA), a dopamine metabolite, in urine has been proposed as an indicator of altered dopamine metabolism in PCOS patients 37. These findings suggest that excessive dopamine expression may contribute to the progression of PCOS (**see Figure 3**), and that modulation of dopamine metabolism may offer a potential therapeutic strategy.

## 4. Management of PCOS and key advances in clinical treatment

Given the significant physical and psychological symptoms associated with PCOS, timely and effective clinical interventions are essential. Beyond conventional nutritional supplementation and pharmacological treatments, addressing the clinical manifestations of PCOS has become a key approach to enhancing the quality of life for affected women 39. In recent years, there is also growing interest in complementary therapies, such as acupuncture and yoga, to alleviate both emotional and physical symptoms in PCOS patients. However, their effectiveness remains unclear and warrants further clinical investigation due to existing limitations and inconsistencies in results. This section provides an overview of current therapeutic approaches and recent clinical advancements in PCOS management, organized by treatment modality.

### 4.1 Pharmacological treatments

In the pharmacological management of PCOS, metformin combined with lifestyle modifications remains the most widely employed strategy, effectively improving menstrual regularity, glycemic control, and lipid profiles 40. Owing to its affordability and favorable safety profile, metformin is extensively utilized, although gastrointestinal discomfort remains a common adverse effect; patients should be adequately counseled to enhance adherence.

For PCOS-associated infertility, clomiphene citrate and letrozole are recommended as first-line agents, shown to improve live birth and clinical pregnancy rates while significantly reducing time-to-pregnancy 41. Among anovulatory PCOS patients, letrozole is the preferred initial therapy. When pharmacological treatment fails, laparoscopic ovarian drilling may be considered to induce ovulation, though it carries inherent risks including anesthesia-related complications, infections, adhesions, and potential adverse outcomes such as multiple gestations or menstrual irregularities 42.

In cases of severe obesity, bariatric surgery offers the most efficacious intervention, rapidly alleviating PCOS-related symptoms; however, pregnancy must be deferred for at least one year postoperatively 43.

### 4.2 Lifestyle and behavioral interventions

Lifestyle interventions, primarily focusing on dietary modifications, increased physical activity, and weight reduction, have been shown to improve free androgen levels; however, no significant benefit has been observed in patients with regular menstruation 12. PCOS-related complications, including obesity, diabetes, and other metabolic disorders, require strict management in accordance with established clinical guidelines. Obese patients should routinely monitor blood pressure and lipid profiles, adhere to statin therapy as prescribed, and engage in regular physical activity to manage body weight effectively 44. For individuals with diabetes or identified high-risk factors, an oral glucose tolerance test is recommended every 1 to 3 years 45.

Psychological well-being must also be addressed, as a substantial proportion of PCOS patients exhibit moderate to severe psychological health concerns 44. In a randomized study involving 68 PCOS patients, participants were allocated to either an intervention group, which underwent a 4-month behavioral modification program (including physical exercise, dietary adjustment, stress management, and stimulus control), or a control group without intervention. After 4 months, the intervention group demonstrated reduced anxiety, enhanced overall health status, and lower depression scores, suggesting that behavioral modifications can significantly improve mental health outcomes in PCOS patients 46.

### 4.3 Complementary approaches

#### Acupuncture and traditional Chinese medicine

Acupuncture, a non-pharmacological treatment, is easy to administer, widely accepted, and extensively utilized both domestically and internationally. In recent years, its application in the management of PCOS has garnered considerable attention 47. Numerous studies have demonstrated that acupuncture can effectively stimulate follicular development and enhance fertility outcomes, which is particularly significant for women with PCOS. Additionally, acupuncture has been shown to alleviate PCOS symptoms by modulating sex hormone secretion and improving ovulation rates 48.

In a clinical trial involving 1,403 PCOS patients, various interventions were tested, including acupuncture combined with moxibustion, clomiphene, and the combination of acupuncture and clomiphene. The results revealed that the acupuncture + clomiphene combination most notably improved endometrial thickness and reduced the incidence of luteinizing unruptured follicle syndrome (LUFS) and ovarian hyperstimulation syndrome (OHSS). Furthermore, acupuncture alone proved most effective in improving ovulation and pregnancy outcomes among PCOS patients. However, the study's limitation was its relatively small sample size, which requires further research to determine the full extent of acupuncture's impact on clinical outcomes 49.

Another study evaluated the therapeutic impact of acupuncture on alleviating clinical symptoms in PCOS. In this study, acupuncture combined with moxibustion was administered as an adjunct to standard PCOS treatment. Compared to the control group, the experimental group exhibited significantly higher pregnancy and ovulation rates, reduced miscarriage and ovarian volume, and improved levels of LH and insulin 50. These results indicate that acupuncture, in combination with moxibustion, effectively corrects hormonal imbalances in PCOS patients. In contrast to pharmacological treatments, acupuncture offers a more convenient and safer alternative. Particularly in PCOS patients with infertility, acupuncture as a supplementary therapy enhances reproductive hormone regulation and improves pregnancy outcomes 51.

#### Gut microbiota modulation

Recent research increasingly indicates that the onset and progression of numerous diseases are closely associated with significant alterations in gut microbiota composition, and that modulation of gut microbiota may partially delay disease progression 52. Furthermore, acupuncture has been shown to modulate the gut microbiota composition in PCOS patients. Wu et al. administered acupuncture combined with clomiphene therapy to obese PCOS patients, with acupuncture sessions conducted three times per week. Changes in sex hormone profiles, glucose and lipid metabolism parameters, and gut microbiota composition were monitored. Compared to the control group, this combined intervention elevated the abundance of Agathobacter faecis while reducing the levels of Erysipelatoclostridium spiroforme, Streptococcus lutetiensis, and Lactococcus lactis. Furthermore, the intervention significantly lowered LH levels and improved insulin resistance in obese PCOS patients 53.

#### α-Lactalbumin supplementation and inositol therapy

Additionally, α-lactalbumin (α-LA), a globular protein secreted by mammary epithelial cells and present in whey, has demonstrated efficacy in ameliorating clinical symptoms of PCOS through modulation of gut microbiota 54. Moreover, α-LA serves as a vital source of bioactive peptides and essential amino acids, such as lysine and tryptophan, which are critical for maintaining intestinal homeostasis in humans 55. By enhancing the intestinal absorption of natural molecules like inositol, α-LA addresses inositol resistance—a common complication among PCOS patients. Given the high prevalence of intestinal dysbacteriosis in PCOS, α-LA administration effectively ameliorates microbial imbalances, mitigates intestinal inflammation, restores gut ecosystem stability, and consequently supports mental health while alleviating clinical symptoms.

In one study, fecal and vaginal samples from PCOS patients were analyzed to assess gut microbiota composition, with α-LA supplementation introduced during microbial cultivation to evaluate its impact on growth capacity. The results demonstrated that α-LA significantly enhanced the proliferation of two beneficial strains, Bifidobacterium and Lactobacillus, both markedly depleted in the intestines and vaginas of PCOS patients. These outcomes further substantiate the roles of α-LA in promoting mental well-being and relieving clinical manifestations in PCOS by modulating gut microbiota homeostasis 56.

Another study enrolled PCOS patients presenting with anovulation and infertility, initially administering oral inositol for three months. For those who did not ovulate, an additional 50 mg of α-LA was introduced alongside ongoing inositol therapy. Subsequent observations on ovulation status and hormone levels were performed. Results indicated that after inositol therapy alone, 23 (62%) achieved ovulation, while 14 remained anovulatory. After addition of α-LA for those with anovulation, 12 (86%) achieved ovulation, accompanied by significant improvements in hormone profiles and blood lipid levels. These results suggest that the combined administration of inositol and α-LA promotes ovulation and enhances fertility outcomes in PCOS patients 57.

## 5. Summary and prospects

The incidence of PCOS among women of reproductive age continues to rise, with lifelong complications imposing a persistent burden on affected individuals and presenting substantial challenges to medical and scientific research communities. Comprehensive investigation into the genetic and pathophysiological mechanisms underlying PCOS remains critical for elucidating its etiology and advancing more effective clinical interventions 6. Since the initial report of PCOS by American physician Irving F. Stein, understanding of the disorder has progressively evolved. However, despite decades of research, significant clinical gaps and unmet needs persist, necessitating urgent attention from the research and clinical communities.

### 5.1 Current clinical gaps and challenges

Several critical gaps in PCOS management warrant immediate attention. First, the heterogeneity in diagnostic criteria remains a major obstacle to consistent clinical practice and research progress. The coexistence of multiple diagnostic frameworks—including the Rotterdam criteria, NIH criteria, and Androgen Excess and PCOS Society criteria—leads to substantial variability in PCOS diagnosis across different healthcare settings and geographic regions 58, 59. This diagnostic inconsistency complicates epidemiological studies, hinders cross-study comparisons, and impedes the development of universal treatment guidelines. Establishing internationally harmonized diagnostic standards is essential for advancing both clinical care and research.

Second, mental health interventions for PCOS remain critically underdeveloped. Despite compelling evidence that 30-60% of PCOS patients experience significant psychological distress, including anxiety, depression, body image dissatisfaction, and infertility-related emotional burden, evidence-based psychological interventions are scarce and poorly integrated into routine PCOS management 44, 46. Current treatment paradigms predominantly focus on reproductive and metabolic manifestations while inadequately addressing the profound psychological impact of the condition. The lack of validated screening tools, standardized mental health assessment protocols, and accessible psychological support services represents a major unmet need. Addressing these psychological issues should be recognized as equally important as managing the physical symptoms of PCOS. Future clinical guidelines must prioritize mental health screening, integrate psychological support into multidisciplinary care teams, and develop tailored cognitive-behavioral interventions specifically designed for PCOS patients.

Third, considerable heterogeneity exists in the clinical manifestations among PCOS patients, ranging from lean phenotypes with minimal metabolic dysfunction to obese presentations with severe insulin resistance and cardiometabolic complications. This phenotypic diversity necessitates individualized treatment approaches; however, current clinical practice often applies standardized protocols that may not adequately address the unique needs of each patient subgroup.

### 5.2 Future research directions and emerging therapeutic strategies

Advancing our understanding of PCOS pathogenesis through genetic and molecular profiling represents a critical frontier for future research. The development of PCOS classification systems based on genetic markers, metabolic phenotypes, and hormonal profiles would enable precision medicine approaches tailored to individual patient characteristics. Genome-wide association studies (GWAS) and multi-omics analyses integrating genomic, transcriptomic, proteomic, and metabolomic data hold promise for identifying novel pathogenic pathways and therapeutic targets. Such molecular stratification could facilitate early risk prediction, particularly in adolescent females, and guide the selection of optimal treatment regimens based on individual genetic and metabolic profiles.

Microbiota-based therapies represent an exciting and rapidly emerging area of investigation. Accumulating evidence implicates gut microbiota dysbiosis in PCOS pathophysiology, suggesting its potential as a novel therapeutic target 52, 53. However, a more detailed understanding of the complex interplay between gut microbiota composition, metabolic regulation, immune responses, and hormonal homeostasis in PCOS is essential. Future research should focus on:

1. Characterizing PCOS-specific microbiome signatures across different phenotypes

2. Elucidating mechanisms by which microbiota influences androgen metabolism, insulin sensitivity, and inflammatory pathways

3. Developing targeted microbiota-based interventions, including probiotics, prebiotics, synbiotics, and fecal microbiota transplantation

4. Conducting large-scale randomized controlled trials to evaluate the efficacy and safety of microbiome-modulating therapies

The integration of artificial intelligence (AI) and advanced computational technologies offers transformative potential for PCOS management. AI-driven algorithms combined with genetic testing and real-time metabolic monitoring could optimize ovulation induction strategies, predict treatment responses, minimize risks such as ovarian hyperstimulation syndrome, and enhance live birth rates. Machine learning models analyzing multi-dimensional clinical, biochemical, and imaging data may improve diagnostic accuracy, enable automated phenotype classification, and facilitate personalized treatment recommendations.

Additional research priorities include:

1. Investigation of neuroendocrine mechanisms underlying GnRH dysregulation and development of targeted neuromodulatory therapies

2. Exploration of anti-inflammatory agents and antioxidants to address chronic low-grade inflammation

3. Examination of epigenetic modifications and their roles in transgenerational transmission of PCOS risk

4. Development of novel biomarkers for early diagnosis, disease monitoring, and treatment response prediction

Optimizing clinical management and multidisciplinary care

In therapeutic practice, given the marked variability among patients, the formation of multidisciplinary medical teams—including endocrinologists, gynecologists, reproductive specialists, dietitians, psychologists, and mental health professionals—is strongly recommended to design individualized treatment strategies. This integrated approach enables more effective disease management and improves patients' quality of life and emotional well-being.

Pharmacological interventions for PCOS have been implemented in clinical practice for decades, and current therapeutic regimens are relatively well-established, with treatment strategies typically selected based on clinical guidelines 60. Metformin and letrozole represent established first-line therapeutic options; however, given the considerable heterogeneity among PCOS patients, individualized treatment regimens must be tailored based on specific clinical profiles, including optimal dosing, treatment duration, combination strategies, and safety considerations. For PCOS patients with infertility, optimizing ovulation induction through integration of pharmacological interventions, acupuncture, and emerging technologies is essential to improve reproductive outcomes.

Early diagnosis, precise phenotypic classification, and stratified treatment should be prioritized, particularly in adolescent females, to prevent long-term complications and improve life-course health trajectories. Establishing PCOS classification criteria based on genetic and metabolic profiles would enable the development of truly personalized therapeutic regimens. Timely identification of long-term cardiometabolic complications through regular screening and implementation of targeted lifestyle modifications could further contribute to improved patient outcomes.

Complementary therapies, including acupuncture, have demonstrated promising results in improving ovulation rates and hormonal balance, offering safe and accessible options that are increasingly accepted by patients 47-51. Evidence suggests that acupuncture may also beneficially modulate gut microbiota composition 53, highlighting potential synergistic mechanisms. Continued investigation into these integrative approaches is warranted.

## Conclusions

PCOS represents a complex, multifaceted disorder requiring comprehensive approaches that address reproductive, metabolic, and psychological dimensions. While significant progress has been made in understanding its pathophysiology and expanding treatment options, critical gaps remain—particularly in mental health support, diagnostic standardization, and personalized medicine implementation. Future research should prioritize elucidating pathogenic mechanisms through advanced molecular profiling, developing microbiota-based and AI-integrated therapies, and establishing evidence-based psychological interventions. Early diagnosis, phenotype-specific treatment strategies, and holistic multidisciplinary care models are essential for optimizing patient outcomes. Through continued innovation, international collaboration, and commitment to addressing both the physical and psychological burdens of PCOS, the medical and scientific communities can substantially enhance reproductive health, mental well-being, and overall quality of life for millions of affected women globally.

## Figures and Tables

**Table 1:** Pathogenic Factors of PCOS.

Pathogenic Factor	Description	References
Hyperandrogenism	Excessive accumulation of androgens leads to ovarian dysfunction, affects normal follicular development, and results in polycystic ovaries.	14, 15.
Insulin Resistance	Elevated insulin levels stimulate LH receptor expression, leading to an imbalanced LH/FSH ratio, affecting follicular development. Insulin also exacerbates fat accumulation, worsening endocrine disorders.	16
Obesity (Especially Visceral Fat)	Visceral fat accumulation leads to hyperlipidemia and hyperinsulinemia, which in turn causes imbalanced LH/FSH ratio and affects normal follicular development.	15
Leptin Abnormality	Leptin inhibits the expression of aromatase, reducing the conversion of androgens to estrogens, leading to excessive androgen accumulation.	17
ADAMTS-1 Metalloproteinase Abnormality	Abnormal expression of ADAMTS-1 metalloproteinase affects follicular rupture and oocyte release, promoting polycystic ovary formation.	18
GnRH Pulsatile Secretion Abnormality	Overactivity of GnRH neurons leads to excessive LH secretion, forming a self-reinforcing pathological feedback loop, disrupting fertilization and embryo development.	21, 22
Kisspeptin Abnormality	Kisspeptin upregulates GnRH release, promoting increased LH and androgen secretion, and is a key factor in the progression of PCOS.	23, 24.
Acetylcholine Metabolism Abnormality	Reduced acetylcholine responsiveness may lead to gastroparesis and impaired gastrointestinal motility.	27-29
Dopamine Metabolism Abnormality	Abnormal dopamine levels may exacerbate PCOS progression, affecting ovarian function, possibly through interactions between dopamine receptors and GnRH and LH, leading to endocrine imbalance.	35, 38
